# pH-Responsive Self-Assembly of Designer Aromatic Peptide Amphiphiles and Enzymatic Post-Modification of Assembled Structures

**DOI:** 10.3390/ijms22073459

**Published:** 2021-03-27

**Authors:** Rie Wakabayashi, Ayato Higuchi, Hiroki Obayashi, Masahiro Goto, Noriho Kamiya

**Affiliations:** 1 Department of Applied Chemistry, School of Engineering, Kyushu University, Fukuoka 819-0395, Japan; higuchi.ayato.808@s.kyushu-u.ac.jp (A.H.); obayashi.hiroki.495@s.kyushu-u.ac.jp (H.O.); m-goto@mail.cstm.kyushu-u.ac.jp (M.G.); 2 Center for Future Chemistry, Kyushu University, Fukuoka 819-0395, Japan

**Keywords:** self-assembly, peptide amphiphile, enzymatic reaction, pH-responsiveness, post-modification

## Abstract

Supramolecular fibrous materials in biological systems play important structural and functional roles, and therefore, there is a growing interest in synthetic materials that mimic such fibrils, especially those bearing enzymatic reactivity. In this study, we investigated the self-assembly and enzymatic post-modification of short aromatic peptide amphiphiles (PAs), Fmoc-L_n_QG (*n* = 2 or 3), which contain an LQG recognition unit for microbial transglutaminase (MTG). These aromatic PAs self-assemble into fibrous structures via π-π stacking interactions between the Fmoc groups and hydrogen bonds between the peptides. The intermolecular interactions and morphologies of the assemblies were influenced by the solution pH because of the change in the ionization states of the C-terminal carboxy group of the peptides. Moreover, MTG-catalyzed post-modification of a small fluorescent molecule bearing an amine group also showed pH dependency, where the enzymatic reaction rate was increased at higher pH, which may be because of the higher nucleophilicity of the amine group and the electrostatic interaction between MTG and the self-assembled Fmoc-L_n_QG. Finally, the accumulation of the fluorescent molecule on these assembled materials was directly observed by confocal fluorescence images. Our study provides a method to accumulate functional molecules on supramolecular structures enzymatically with the morphology control.

## 1. Introduction

Supramolecular fibrils formed through molecular self-assembly are abundant in biological systems; examples include extracellular collagen matrices, intracellular actin filaments, and microtubules. They play important structural and functional roles. Synthetic approaches to fabricate materials that mimic such fibrils have been developed using various molecules [1,2]. Peptide amphiphiles (PAs) are one promising class of synthetic molecules used to fabricate such fibril-mimicking materials because of their design diversity and bio-functionality [3,4,5,6,7,8]. Artificial extracellular matrices, as well as drug delivery carriers with varied morphologies, mechanical properties, and biological epitopes have been reported thus far.

One important feature of these supramolecular materials is their responsiveness to external stimuli [9,10]. Since supramolecular organizations are dependent on relatively weak noncovalent interactions, they can be susceptible to external stimuli, such as light, temperature, and pH. PAs often contain pH-responsive side chains or an N- or C-terminus; therefore, pH is one of the most common stimuli used to control the assembly [11,12]. Indeed, many studies have shown pH-induced changes in morphology [13] and mechanical properties [14], which resulted in functional materials, such as drug-releasing capsules [15,16] and injectable cell delivery vehicles [14].

The use of enzymatic reactions to control morphologies or assembly formation is a relatively new concept in supramolecular materials [17,18]. Here, the chemical conversion of molecular structures is induced by an enzyme. There is a growing interest in such systems not only because of their analogy to biological systems, but also their controllability under biocompatible conditions. Various enzymes, including proteases [19,20], phosphatases [21], kinases [22], and tyrosinases [23] have been used to demonstrate this concept. In contrast, post-modification of supramolecular fibrils by enzymatic reactions is limited to a few reports [24,25,26]. This strategy contributes to adding functions or controlling hierarchical structures, or both, in biological systems [27,28]; however, there are limited number of enzymes capable of performing this task. Cross-linking enzymes, such as transglutaminase [29] and sortase [30], are some of the few limited enzymes.

We have previously reported on novel short aromatic peptide amphiphiles, 9-fluorenylmethoxycarbonyl-(Leu)_n_-Gln-Gly (Fmoc-L_n_QG, *n* = 2 or 3) [25]. These PAs contain a microbial transglutaminase (MTG)-reactive Gln residue. MTG catalyzes cross-linking reactions between the γ-carboxyamide of Gln and primary amines; thus Fmoc-L_n_QG enables post-modification of the self-assembly structures with amine-containing molecules by the MTG reaction. A significant achievement from this work was that the post-modification was performed on different self-assembled structures, and the structure was depending on the number of Leu residues: specifically, narrow fibrils were formed by Fmoc-L_2_QG, and wide, flat tapes from Fmoc-L_3_QG.

**Figure 1:**
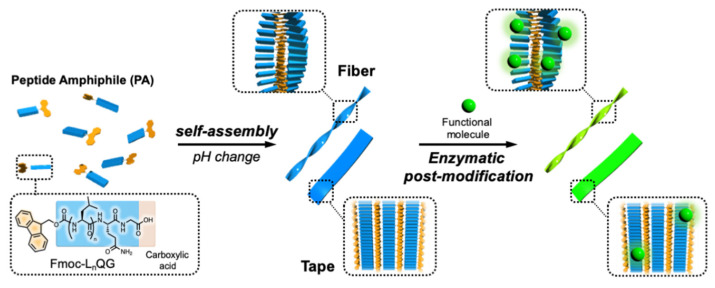
Conceptual diagram of this study. Short aromatic peptide amphiphiles bearing enzymatic reaction sites first self-assemble into different structures, then post-modification of the structures was achieved by the enzymatic reaction.

In this study, we focused on the pH responsiveness of these assemblies. Since the free acid of the C-terminus shows different ionization states depending on the pH, the self-assembly of Fmoc-L_n_QG should be influenced by the pH. We first investigated the self-assembly of Fmoc-L_n_QG at four different pH values from pH 5 to 8. We then evaluated the enzymatic reaction on the Fmoc-L_n_QG assemblies at various pH values. Our results showed that both the self-assembly and enzymatic reaction are highly dependent on the pH, while both were maintained (Figure 1).

## 2. Results and Discussion

### 2.1. Change in Self-Organization Behavior of Fmoc-L_n_QG (n = 2, 3) Depending on pH

#### 2.1.1. Evaluation of Interaction between Fmoc Groups by Fluorescence Spectroscopy

**Figure 2:**
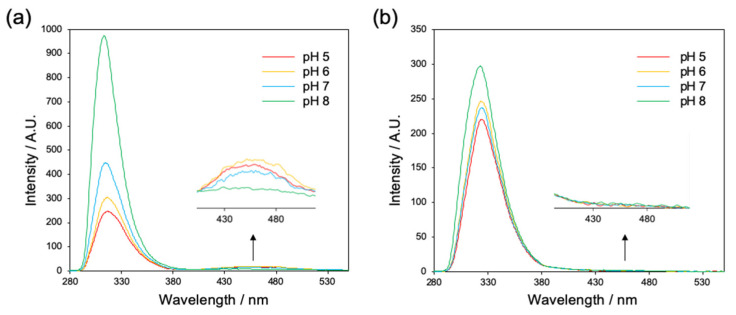
Fluorescence spectra of Fmoc-L_2_QG (**a**) and Fmoc-L_3_QG (**b**) at pH 5–8 above their critical aggregation concentrations (CACs). λ_ex_ = 265 nm.

Since the aromatic stacking interactions of short aromatic peptides have a large impact on the self-organization, we first evaluated the interaction between the Fmoc groups of Fmoc-L_n_QG (*n* = 2, 3) at pH 5–8 by fluorescence spectroscopy below and above the critical aggregation concentrations (CAC values were 0.14, 0.20, 0.092, and 0.53 mM for Fmoc-L_2_QG and 0.089, 0.041, 0.14, and 0.091 mM for Fmoc-L_3_QG at pH 5, 6, 7, and 8, respectively: Figure S2). For Fmoc-L_2_QG in a monomeric state (0.005 mM), the maximum monomeric fluorescence was observed at 316 nm (Figure S3a). The peaks were shifted to 318 nm in an assembled state (2.0 mM; Figure 2a) under all pH conditions (pH 5–8), indicating the presence of excimers of fluorenes [31]. In addition, a broad peak at a longer wavelength around 450 nm was observed (Figure 2a), which corresponded to multiple aromatic stacking interactions (π-π interactions) between the Fmoc groups. While the fluorescence intensity around 450 nm decreased as the pH increased, with a significant decrease at pH 8, the intensity of the excimer fluorescence (~318 nm) increased at higher pH, indicating the change in the π-π interaction mode from long-range to short-range interaction. In Fmoc-L_3_QG, the maximum wavelength of the monomeric fluorescence (314 nm at 0.005 mM) (Figure S3b) was shifted to 324 nm at all pHs when the concentration was above the CAC (Figure 2b). The intensity of the excimer fluorescence increased at higher pH as well. However, there was almost no broad peak around 450 nm. These results suggest that the long-range π-π interaction between Fmoc groups contributes to the higher-order aggregation of Fmoc-L_2_QG, especially at lower pH, while only a short-range interaction between Fmoc groups (excimer formation) was observed for the self-assembled Fmoc-L_3_QG.

#### 2.1.2. Evaluation of Interaction between Peptides by Fourier Transform-Infrared Spectroscopy (FT-IR)

**Figure 3:**
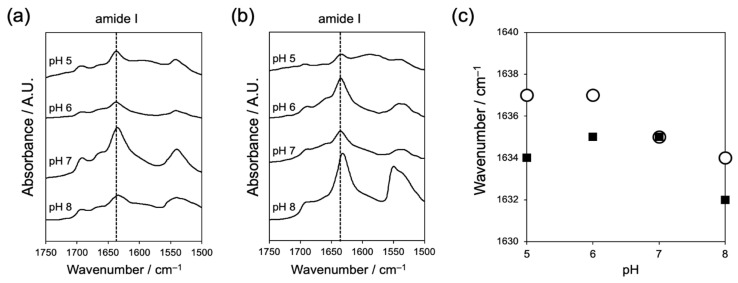
(**a**,**b**) Fourier transform-infrared spectroscopy (FT-IR) spectra of Fmoc-L_2_QG (**a**) and Fmoc-L_3_QG (**b**) at pH 5–8 and (**c**) amide I peak positions of Fmoc-L_2_QG (open circle) and Fmoc-L_3_QG (closed square) at pH 5–8.

The formation of hydrogen bonds between peptides is another important factor in the self-assembly of short aromatic peptides. We used FT-IR to evaluate the effect of pH on the hydrogen bonding between peptides (Figure 3). The amide I band derived from the C = O stretching vibration of the amide groups is an indicator of the strength of hydrogen bonds between peptides. Both Fmoc-L_2_QG and Fmoc-L_3_QG assemblies showed an amide I band around 1635 cm^−1^, indicating the formation of a β-sheet structure (Figure 3a,b). Fmoc-L_3_QG assemblies showed absorption at lower wavenumbers than Fmoc-L_2_QG assemblies. Moreover, the band shifted to a lower wavenumber as the pH increased (Figure 3c). These results indicate that the hydrogen bonds formed between the peptides are stronger in Fmoc-L_3_QG assemblies, especially at higher pH. An additional peak around 1690 cm^−1^ corresponds to the organized carbamate structure of the Fmoc groups.

#### 2.1.3. Evaluation of the Self-Assembled Structure of Fmoc-L_n_QG in Response to pH by Transmission Electron Microscopy (TEM)

**Figure 4:**
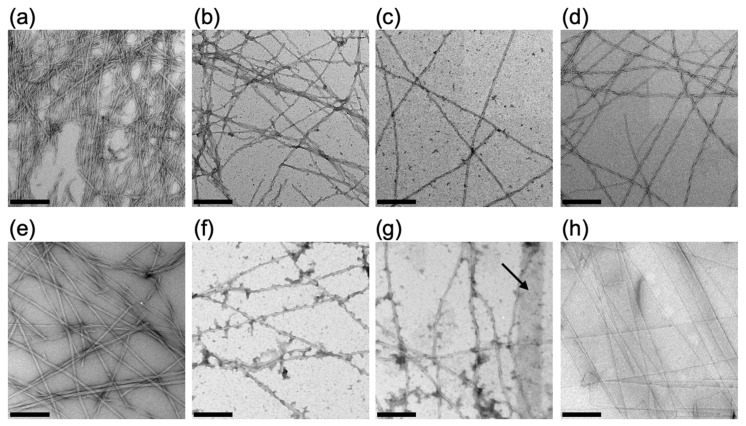
Transmission electron microscopy (TEM) images of Fmoc-L_2_QG (**a**–**d**) and Fmoc-L_3_QG (**e**–**h**) at pH 5 (**a**,**e**), 6 (**b**,**f**), 7 (**c**,**g**), and 8 (**d**,**h**). Fmoc-L_2_QG assemblies formed a narrow fibrous structure with twisting (**a**–**d**). Fmoc-L_3_QG assemblies formed a similar structure at pH 5–6 (**e**,**f**), while they transformed into a wide, flat tape-like structure as the pH increased (**g**,**h**). Arrow in (**g**) indicates the flat tape-like structure. Bars: 200 nm.

We observed how the self-assembled structures of Fmoc-L_n_QG (*n* = 2, 3) changed with pH using transmission electron microscopy (TEM) (Figure 4). Fmoc-L_2_QG assemblies formed narrow fiber-like structures with a twisted morphology at all pH regions between 5 and 8 (Figure 4a–d). The diameter of the fibers remained almost identical (ca. 10 nm), while the twisting was most pronounced at pH 5 (Figure 4a). Similarly, Fmoc-L_3_QG formed fibrous assemblies with a twisted structure at pH 5 and 6 (Figure 4e,f). However, as the pH increases, a wide, flat, tape-like morphology appeared (Figure 4g,h). At pH 8, almost all assemblies were transformed into tape-like structures with a width of ca. 200 nm, which was ca. 12.5-fold wider than those at pH 5 (Figure 4h).

In Fmoc-L_2_QG assemblies, long-range π-π interactions between the Fmoc groups operate, while (relatively) weak hydrogen bonds between the peptides are also in play. In contrast, in Fmoc-L_3_QG assemblies, the π-π stacking interactions are only effective at a short range, and strong hydrogen bond formation is the main driving force of the self-assembly. Although the self-assembled morphologies of Fmoc-L_2_QG at pH 5–8 and Fmoc-L_3_QG at low pH (pH 5 and 6) look similar, the molecular organization may differ. Moreover, Fmoc-L_3_QG at pH 8 showed a drastic morphological change to tape-like structures. This may stem from the additional interaction between the hydrophobic peptide sequence, L_3_, as a result of the stronger hydrogen bond formation, leading to the formation of hierarchical assemblies between fibers.

### 2.2. Enzymatic Modification of Fmoc-L_n_QG (n = 2, 3) Assemblies with Small Fluorescent Substrates

#### 2.2.1. Conjugation of Fmoc-L_n_QG and Oregon Green 488 Cadaverine (OG) by MTG Catalysis

**Figure 5:**
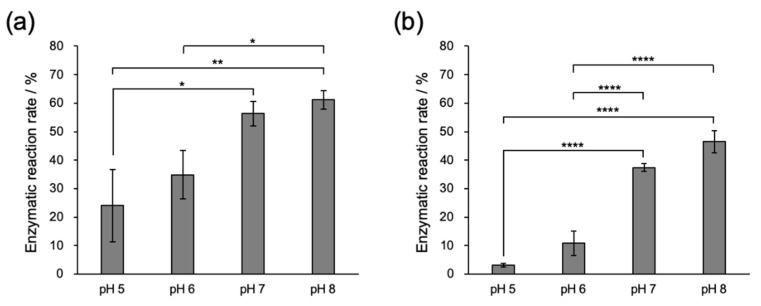
Microbial transglutaminase (MTG) enzymatic reaction rates of (**a**) Fmoc-L_2_QG and (**b**) Fmoc-L_3_QG in an assembled state. The enzymatic reaction was performed with Oregon green 488 cadaverine (OG) as an amine substrate. *n* = 3, * *p* < 0.05, ** *p* < 0.01, **** *p* < 0.0001.

Next, we evaluated the enzymatic reaction rate of MTG using Fmoc-L_n_QG and a small fluorescent substrate with a primary amine, Oregon green 488 cadaverine (OG) at different pHs. Assemblies of Fmoc-L_n_QG were formed, and the MTG reaction with OG was performed at 25 °C for 2 h. The conjugation of OG with Fmoc-L_n_QG was confirmed by Matrix Assisted Laser Desorption/Ionization Time Of Flight Mass Spectrometry (MALDI TOF MS) (Figure S4). Analysis by HPLC indicated that the enzymatic reaction rate increased as the pH increased for both Fmoc-L_n_QG assemblies (Figure 5). Given that the enzymatic activity of MTG measured by the hydroxamate method [32] was almost identical under all pH conditions between pH 5 and 8 (Figure S5a,b), the difference in the reaction rates is a result of the substrates, Fmoc-L_n_QG or OG.

To investigate the influence of the electric charge of the amine substrates, OG was changed to tetramethylrhodamine cadaverine and sulforhodamine cadaverine, which have cationic properties on their aromatic rings. Similarly to OG, the enzyme reaction rates increased at higher pH (Figure S6a–d), suggesting that the net charge of the amine substrate does not directly affect the enzymatic reaction. However, a possibility remains that the pH dependency in the enzymatic rates was because of the amine substrate and its nucleophilicity, which has an intrinsic pH dependency. In fact, initial velocities of the MTG reaction using a simple amine substrate, acetyl-L-lysine, were highly dependent on pH when Fmoc-L_n_QG in an unassembled state or Z-QG was used as the glutamine substrate (Figures S7 and S8).

In the case of the assembled glutamine substrate, Fmoc-L_n_QG assemblies, we evaluated the apparent pKa values by a titration method. Although the theoretical pKa value of the carboxy group of a C-terminal amino acid of Fmoc-L_n_QG, glycine is ca. 3.5, the value can shift because of the influence of neighboring molecules in an assembled state [31]. The apparent pKa values indeed shifted to 6.2–8.1 and 5.8–7.9 for Fmoc-L_2_QG and Fmoc-L_3_QG, respectively, with the inflection point around 7 (Figure S9). Considering that the pI of MTG is 8.9, the accessibility of MTG to Fmoc-L_n_QG assemblies may increase above pH 7. In addition, enhanced hydrogen bonding between the peptides at higher pH (Figure 3) increases the apparent concentration of the Gln substrate at the enzymatic reaction site, which may increase the affinity to MTG. Taken together, higher pH optimized the environment for the enzymatic reaction for both the amine and assembled glutamine substrates, which results in the higher reaction rates found in Figure 5.

#### 2.2.2. Confocal Fluorescence Microscope Images of OG on Fmoc-L_n_QG Peptide Assemblies

**Figure 6:**
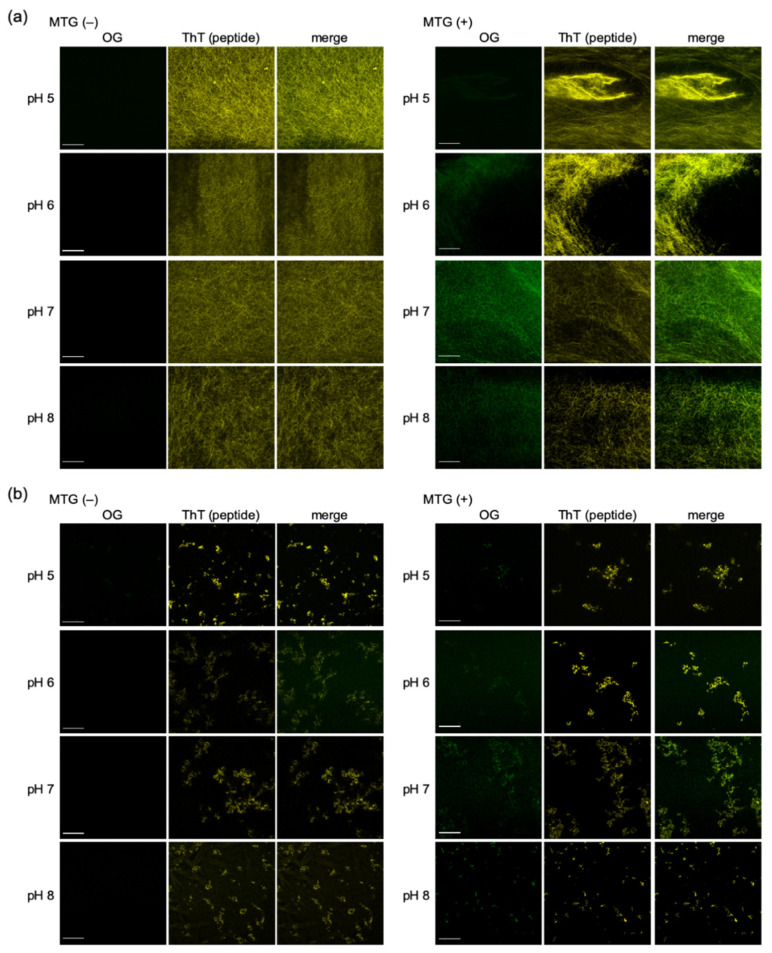
Confocal laser scanning microscopy (CLSM) images of Fmoc-L_2_QG (**a**) and Fmoc-L_3_QG (**b**) assemblies at pH 5–8 with and without the MTG reaction. Green fluorescence derived from Oregon green 488 cadaverine (OG) was observed at the overlap region with the fluorescence of thioflavin T (ThT)-stained peptide amphiphile (PA) assemblies for samples with the MTG reaction. Bars: 20 μm.

Finally, we confirmed the accumulation of OG on the Fmoc-L_n_QG assemblies by observation with confocal laser scanning microscopy (CLSM) [33]. In both PA assemblies, green fluorescence from OG was observed at the overlap region of thioflavin T (ThT)-stained PA assemblies after the MTG reaction (Figure 6a,b; right panels). In contrast, in the control samples without an MTG reaction, no fluorescence from OG was observed on the PA assemblies (Figure 6a,b; left panels). Despite the low reaction rates at low pH, especially for Fmoc-L_3_QG (Figure 5), a sharp contrast in the fluorescence of OG between with (right panels) and without (left panels) MTG reaction samples was observed. The contrast was more obvious with samples that showed high reaction rates, such as Fmoc-L_2_QG at pH 7 and 8, where the green fluorescence derived from OG was uniformly found throughout the peptide assembly structures (Figure 6a, right panels). These results suggest that the specific accumulation of OG on the Fmoc-L_n_QG assemblies was achieved by the MTG reaction under all the pH conditions examined, though the accumulation ratio depended on the reaction rates, and the accumulation was directly observed using a CLSM technique.

## 3. Experimental Section

### 3.1. General

Amino acid reagents and resin for the synthesis of PA, Fmoc-Gly-Alko-resin, Fmoc-Leu-OH, Fmoc-Gln(Trt)-OH, O-(1H-benzotriazol-1-yl)-N,N,N′,N′-tetramethyluronium hexafluorophosphate (HBTU), 1-hydroxy-1-H-benzotriazole hydrate (HOBt), N,N-diisopropylethylamine (DIEA), piperidine, trifluoroacetic acid (TFA), Nα-acetyl-L-lysine(acetyl-L-Lys), and triisopropylsilane (TIS) were purchased from Watanabe Chemical Industries (Hiroshima, Japan). Reagents for the Kaiser test were purchased from Kokusan Chemical (Tokyo, Japan). Methanol, dichloromethane, diethyl ether, acetonitrile (ACN), Nile red, sodium hydrogenphosphate dodecahydrate, and trizma base were obtained from Wako Pure Chemical Industries (Osaka, Japan). N,N-dimethylformamide (DMF), N-ethylmaleimide (NEM), dimethyl sulfoxide (DMSO), triethylamine (TEA), acetic acid (AcOH), citric acid, sodium dihydrogenphosphate dihydrate, and hydrochloric acid were purchased from Kishida Chemical (Osaka, Japan). Oregon green 488 cadaverine, fluorescein cadaverine, and sulforhodamine cadaverine were purchased from Thermo Fisher Scientific (Waltham, MA, USA). Tetramethylrhodamine cadaverine was purchased from Cosmo Bio (Tokyo, Japan). Thioflavin T (ThT) was obtained from Sigma Aldrich (St. Louis, MO, USA). Trifluoroethanol (TFE) was obtained from Tokyo Chemical industry (Tokyo, Japan). Tri-sodium citrate dihydrate was purchased from Nacalai Tesque (Kyoto, Japan). All chemicals and solvents were used as received. In this study, the following buffer solutions were used: sodium citrate buffer for pH 5, phosphate buffer for pH 6 and 7, and Tris-HCl buffer for pH 8. The concentrations of buffer were set to 10 mM except for that shown in Figure S5b.

### 3.2. Synthesis of Aromatic Peptide Amphiphiles

Fmoc-L_n_QG (*n* = 2, 3) were synthesized by the standard 9-fluorenylmethoxycarbonyl (Fmoc) solid-phase peptide synthesis method. Fmoc-Gly-Alko Resin was immersed in dichloromethane for 30 min. The protective Fmoc group was removed using 20% piperidine in DMF. The deprotection was confirmed by Kaiser tests. Coupling reactions of each amino acid were conducted by adding a mixture of coupling reagents (Fmoc-amino acid:HBTU:HOBt:DIEA = 3:3:3:6 mol equivalent to reactive sites on resin) in DMF to the resin and shaken for 1 h. After coupling reactions, the protective Fmoc group was removed by 20% piperidine in DMF except for the last amino acid. The aromatic peptides were cleaved from the resin using a mixture of 95% TFA, 2.5% TIS, and 2.5% water for 1.5 h. After removing the solvents under reduced pressure, the peptides were precipitated and washed with cold diethyl ether.

The crude peptide solids were collected, dissolved in the mixture of DMSO, TFE, and a 0.1% aqueous solution of TEA/AcOH (11:2 (*v*/*v*)) with 2:2:6 (*v*/*v*/*v*) ratio, and purified by reverse-phase high pressure liquid chromatography (HPLC) on Inertsil ODS-3 column (GL Science, Tokyo, Japan) using a gradient of water and acetonitrile both containing 0.1% TEA/AcOH. The fractions with each PA were collected, lyophilized, and stored at −20 °C until use. The purified peptides were analyzed by HPLC (Inertsil ODS-3 column, GL science, Tokyo, Japan) and MALDI TOF MS (Autoflex-III, Bruker, Billerica, MA, USA) using α-cyano-4-hydroxycinnamic acid (CHCA, Sigma-Aldrich (St. Louis, MO, USA)) as the matrix.

### 3.3. Preparation of MTG

MTG was recombinantly prepared in *Escherichia coli* BL21 star (DE3) as previously reported [34]. Briefly, a chimera protein of maltose-binding protein and tobacco etch virus protease (MBP-TEV) was fused to the N-terminus of *Streptomyces mobaraensis* MTG. The TEV protease recognition sequence (ENLFYQS) was inserted between the propeptide domain and the catalytic domain of MTG. Two mutations, K10R and Y12A, were introduced to the propeptide domain. The active MTG without the propeptide domain was prepared by a self-cleavage reaction of the MBPTEV-propeptide-MTG. The expressed active MTG was purified with a NiNTA column (HisTrap FF Crude, 5 mL, Cytiva, Tokyo, Japan), and a size-exclusion column (HiLoad 16/600 Superdex 75 pg, Cytiva) using standard protocols. The amino acid sequence of the active MTG prepared is shown below.

SGGGGSDSDDRVTPPAEPLDRMPDPYRPSYGRAETVVNNYIRWQQVYSHRDGRKQQ
MTEEQREWLSYGCVGVTWVNSGQYPTNRLAFASFDEDRFKNELKNGRPRSGETRAE
FEGRVAKESFDEEKGFQRAREVASVMNRALENAHDESAYLDNLKKELANGNDALR
NEDARSPFYSALRNTPSFKERNGGNHDPSRMKAVIYSKHFWSGQDRSSSADKRKYGD
PDAFRPAPGTGLVDMSRDRNIPRSPTSPGEGFVNFDYGWFGAQTEADADKTVWTHG
NHYHAPNGSLGAMHVYESKFRNWSEGYSDFDRGAYVITFIPKSWNTAPDKVKQGW
P

### 3.4. Critical Aggregation Concentration (CAC)

Various concentrations of Fmoc-L_n_QG (*n* = 2, 3) samples were prepared in 10 mM buffer at pH 5–8. For each peptide solution, the fluorescent dye, Nile red, was added at a final concentration of 1 μM and incubated overnight at room temperature. The fluorescence intensity at 635 nm (excitation wavelength 560 nm) of each sample was measured using a microplate reader (SpectraMax i3x, Molecular Device, San Jose, CA, USA) and plotted against the peptide concentration to create a CAC plot.

### 3.5. Fluorescence Spectra

Fmoc-L_2_QG (0.005 or 2.0 mM) and Fmoc-L_3_QG (0.005 or 1.0 mM) were prepared as the self-assembled (2.0 and 1.0 mM for Fmoc-L_2_QG and Fmoc-L_3_QG, respectively) or un-assembled (0.005 mM) samples. Fluorescence spectra were acquired from 270 to 550 nm by exciting at 265 nm. Fluorescence spectra were measured using an LS55 fluorescence spectrometer (PerkinElmer, Waltham, MA, USA).

### 3.6. Fourier-Transform Infrared Spectroscopy (FT-IR)

Fmoc-L_2_QG (2.0 mM) and Fmoc-L_3_QG (1.0 mM) were prepared in 10 mM buffer at pH 5–8 and lyophilized. Fourier-transform infrared (FT-IR) spectra were recorded on Spectrum Two (PerkinElmer) in ATR mode. A resolution of 2 cm^−1^ was used.

### 3.7. Transmission Electron Microscopy (TEM)

Fmoc-L_2_QG (2.0 mM) and Fmoc-L_3_QG (1.0 mM) were prepared in 10 mM buffer at pH 5–8. Three microliters of each sample were drop-cast onto a hydrophilized STEM grid with an elastic carbon film (Okenshoji, Tokyo, Japan). After 1.5 min of incubation, the excess solution was removed and then stained with 2% uranyl solution for 2 min. The transmission electron microscopy (TEM) images were taken by JEM-2010 (JEOL, Tokyo, Japan) with an accelerating voltage of 120 kV.

### 3.8. Conjugation of Fmoc-L_n_QG and Oregon Green Cadaverine (OG) by MTG Catalysis

A reaction sample of each self-assembled PA was prepared in 10 mM buffer at each pH ([Fmoc-L_2_QG] = 2.0 mM, [Fmoc-L_3_QG] = 1.0 mM, Fmoc-L_n_QG:OG = 10:1 (mol:mol)). MTG (0.3 U/mL) was added to the samples, and the reaction was allowed to proceed at 25 °C for 2 h. After the reaction, NEM at a final concentration of 1 mM was added to inactivate the MTG. The enzymatic reaction rate at each pH was evaluated by HPLC analysis on an Inertsil ODS-3 (4.6 × 250 nm) column. The gradient was from 40% to 80% with 0.1% TFA ACN solution, and the flow rate was 1 mL/min. The OG-containing-eluents were detected at 488 nm.

### 3.9. Confocal Fluorescence Microscope Images of OG on Fmoc-L_n_QG Peptide Assemblies

An aqueous solution of ThT at a final concentration of 10 µM was added to the reaction samples. The droplets of samples (2.5 µL) were transferred into multi-well glass-bottom dishes (Matsunami Glass Ind., Osaka, Japan), and 2.5 µL of 10 mM CaCl_2_ solution was added. The confocal images were taken using LSM700 (Carl Zeiss, Oberkochen, Germany) with diode lasers (405 nm for ThT, 488 nm for OG).

## 4. Conclusions

In this study, we used two short aromatic peptide amphiphiles with MTG reactivity, Fmoc-L_2_QG and Fmoc-L_3_QG, to examine the pH responsiveness of their self-assembly and enzymatic reactions. These PAs self-assemble via π-π stacking interactions between the Fmoc groups and hydrogen bonds between peptides. The intermolecular interactions were influenced by pH; a change in π-π stacking mode from long-range to short-range interaction and an increase in hydrogen bonding were observed when the solution pH was increased from pH 5 to 8. A dramatic morphological change was observed for Fmoc-L_3_QG from twisted fibers at pH 5 to wide, flat, tape-like structures at pH 8. In the post-modification of a small fluorescent substrate, Oregon green 488 cadaverine, on these Fmoc-L_n_QG assemblies, the rate of modification increased at higher pH, presumably because of the higher nucleophilicity of the amine group and increased accessibility of MTG to the assembled Gln substrates. Finally, direct observation of the accumulation of OG on the Fmoc-L_n_QG assemblies was achieved using CLSM. Our study demonstrates the functionalization of supramolecular fibrous materials while also controlling their supramolecular structures. This will provide a new strategy to engineer functional biomimetic nanomaterials for various applications in the biomedical field.

## Supplementary Materials

Supplementary Materials can be found at https://www.mdpi.com/article/10.3390/ijms22073459/s1.

## Abbreviations

MTG	Microbial transglutaminase
CAC	Critical aggregation concentration
FT-IR	Fourier transform infrared spectroscopy
TEM	Transmission electron microscopy
OG	Oregon green 488 cadaverine
CLSM	Confocal laser scanning microscopy

## References

[1] Webber M.J., Appel E.A., Meijer E.W., Langer R. (2015). Supramolecular biomaterials. Nat. Mater..

[2] Aida T., Meijer E.W., Stupp S.I. (2012). Functional supramolecular polymers. Science.

[3] Li J., Xing R., Bai S., Yan X. (2019). Recent advances of self-assembling peptide-based hydrogels for biomedical applications. Soft Matter.

[4] Sato K., Hendricks M.P., Palmer L.C., Stupp S.I. (2018). Peptide supramolecular materials for therapeutics. Chem. Soc. Rev..

[5] Hauser C.A.E., Zhang S. (2010). Designer self-assembling peptide nanofiber biological materials. Chem. Soc. Rev..

[6] Hamley I.W. (2011). Self-assembly of amphiphilic peptides. Soft Matter.

[7] Cui H., Webber M.J., Stupp S.I. (2010). Self-assembly of peptide amphiphiles: From molecules to nanostructures to biomaterials. Biopolymers.

[8] Inaba H., Matsuura K. (2019). Peptide Nanomaterials Designed from Natural Supramolecular Systems. Chem. Rec..

[9] Mart R.J., Osborne R.D., Stevens M.M., Ulijn R.V. (2006). Peptide-based stimuli-responsive biomaterials. Soft Matter.

[10] Shigemitsu H., Hamachi I. (2017). Design Strategies of Stimuli-Responsive Supramolecular Hydrogels Relying on Structural Analyses and Cell-Mimicking Approaches. Acc. Chem. Res..

[11] Ghosh G., Barman R., Sarkar J., Ghosh S. (2019). pH-Responsive Biocompatible Supramolecular Peptide Hydrogel. J. Phys. Chem. B.

[12] Sarkar A., Kölsch J.C., Berač C.M., Venugopal A., Sasmal R., Otter R., Besenius P., George S.J. (2020). Impact of NDI-Core Substitution on the pH-Responsive Nature of Peptide-Tethered Luminescent Supramolecular Polymers. Chem. Open.

[13] Gao C., Li H., Li Y., Kewalramani S., Palmer L.C., Dravid V.P., Stupp S.I., Olvera De La Cruz M., Bedzyk M.J. (2017). Electrostatic Control of Polymorphism in Charged Amphiphile Assemblies. J. Phys. Chem. B.

[14] Tang J.D., Mura C., Lampe K.J. (2019). Stimuli-Responsive, Pentapeptide, Nanofiber Hydrogel for Tissue Engineering. J. Am. Chem. Soc..

[15] Li X., Fu M., Wu J., Zhang C., Deng X., Dhinakar A., Huang W., Qian H., Ge L. (2017). pH-sensitive peptide hydrogel for glucose-responsive insulin delivery. Acta Biomater..

[16] Ahlers P., Frisch H., Holm R., Spitzer D., Barz M., Besenius P. (2017). Tuning the pH-Switch of Supramolecular Polymer Carriers for siRNA to Physiologically Relevant pH. Macromol. Biosci..

[17] Williams R.J., Mart R.J., Ulijn R.V. (2010). Exploiting biocatalysis in peptide self-assembly. Biopolymers.

[18] He H., Tan W., Guo J., Yi M., Shy A.N., Xu B. (2020). Enzymatic Noncovalent Synthesis. Chem. Rev..

[19] Hirst A.R., Roy S., Arora M., Das A.K., Hodson N., Murray P., Marshall S., Javid N., Sefcik J., Boekhoven J. (2010). Biocatalytic induction of supramolecular order. Nat. Chem..

[20] He H., Guo J., Lin X., Xu B. (2020). Enzyme-Instructed Assemblies Enable Mitochondria Localization of Histone H2B in Cancer Cells. Angew. Chem. Int. Ed..

[21] Feng Z., Han X., Wang H., Tang T., Xu B. (2019). Enzyme-Instructed Peptide Assemblies Selectively Inhibit Bone Tumors. Chem.

[22] Yang Z., Liang G., Wang L., Xu B. (2006). Using a kinase/phosphatase switch to regulate a supramolecular hydrogel and forming the supramolecular hydrogel in vivo. J. Am. Chem. Soc..

[23] Gao J., Zheng W., Kong D., Yang Z. (2011). Dual enzymes regulate the molecular self-assembly of tetra-peptide derivatives. Soft Matter.

[24] Collier J.H., Messersmith P.B. (2003). Enzymatic Modification of Self-Assembled Peptide Structures with Tissue Transglutaminase. Bioconjug. Chem..

[25] Wakabayashi R., Suehiro A., Goto M., Kamiya N. (2019). Designer aromatic peptide amphiphiles for self-assembly and enzymatic display of proteins with morphology control. Chem. Commun..

[26] Ohshima T., Sakono M. (2017). Enzymatic Installation of Functional Molecules on Amyloid-Based Polymers. Bioconjug. Chem..

[27] Varland S., Vandekerckhove J., Drazic A. (2019). Actin Post-translational Modifications: The Cinderella of Cytoskeletal Control. Trends Biochem. Sci..

[28] Song Y., Brady S.T. (2015). Post-translational modifications of tubulin: Pathways to functional diversity of microtubules. Trends Cell Biol..

[29] Strop P. (2014). Versatility of Microbial Transglutaminase. Bioconjug. Chem..

[30] Popp M.W.L., Ploegh H.L. (2011). Making and breaking peptide bonds: Protein engineering using sortase. Angew. Chem. Int. Ed..

[31] Tang C., Ulijn R.V., Saiani A. (2011). Effect of glycine substitution on Fmoc-diphenylalanine self-assembly and gelation properties. Langmuir.

[32] Folk J.E., Cole P.W. (1966). Mechanism of Action of Guinea Pig Liver Transglutaminase. J. Biol. Chem..

[33] Kubota R., Nakamura K., Torigoe S., Hamachi I. (2020). The Power of Confocal Laser Scanning Microscopy in Supramolecular Chemistry: In situ Real-time Imaging of Stimuli-Responsive Multicomponent Supramolecular Hydrogels. Chem. Open.

[34] Sato R., Minamihata K., Ariyoshi R., Taniguchi H., Kamiya N. (2020). Recombinant production of active microbial transglutaminase in E. coli by using self-cleavable zymogen with mutated propeptide. Protein Expr. Purif..

