# Supplementary material for: pH-Responsive Self-Assembly of Designer Aromatic Peptide Amphiphiles and Enzymatic Post-Modification of Assembled Structures
Source: Int J Mol Sci. 2021 Mar 27;22(7):3459. doi: 10.3390/ijms22073459 (PMC8037177; doi:10.3390/ijms22073459)
Supplement: Supplementary file 1 [file ijms-22-03459-s001.pdf]

Supplementary Information

Table of Contents

1. Characterization of Fmoc-LnQG peptides S2
2. Critical aggregation concentration determination of Fmoc-L$_n$QG peptides at pH 5-8 S3
3. Fluorescence spectra of Fmoc-L$_2$QG and Fmoc-L$_3$QG at pH 5-8 below CAC S4
4. Evaluation of integration of Oregon green 488 cadaverine (OG) on Fmoc-L$_n$QG peptide assemblies by MALDI-TOF-MS S5
5. Evaluation of MTG specific activity at various pH S7
6. MTG reaction of Fmoc-L$_n$QG peptides with TAMRA cadaverine and Sulforhodamine cadaverine S8
7. MTG reaction rate of Fmoc-L$_n$QG with Ac-Lys-OH at concentrations below CAC S9
8. MTG reaction rate of Z-QG with Ac-Lys-OH S10
9. Titration curves of Fmoc-L$_n$QG assemblies to determine apparent pKa values S11

1. Characterization of Fmoc-L$_n$QG peptides.

The synthesized Fmoc-L$_n$QG peptides were analyzed by MALDI TOF MS and HPLC as shown in **Figure S1**. The major peaks found in MALDI TOF MS spectra corresponded to the sodium adducts (m/z 674.094 and 787.024 for Fmoc-L$_2$QG and Fmoc-L$_3$QG, respectively) and the potassium adducts (m/z 690.094 and 803.010 for Fmoc-L$_2$QG and Fmoc-L$_3$QG, respectively). The purities of Fmoc-L$_2$QG and Fmoc-L$_3$QG were 98.7 % and 98.1 %, respectively, by HPLC analysis.

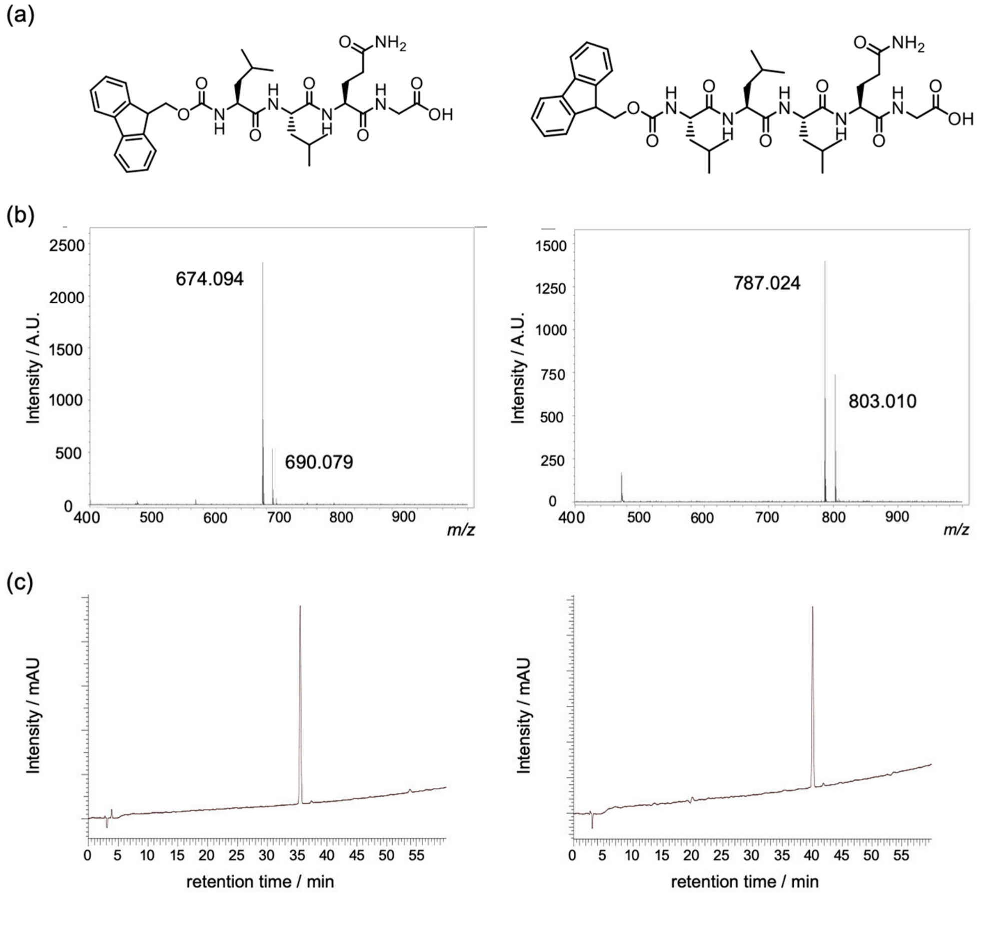

Figure S1. (a) Chemical structures, (b) MALDI TOF MS spectra, and (c) HPLC chromatograms of Fmoc-L$_2$QG (left) and Fmoc-L$_3$QG (right).

2. Critical aggregation concentration determination of Fmoc-L$_n$QG peptides at pH 5-8.

The critical aggregation concentration (CAC) plots were prepared using Nile red-derived fluorescence intensity at 635 nm (**Figure S2**). The CACs of Fmoc-L$_2$QG were 0.14, 0.20, 0.092, and 0.53 mM at pH 5, 6, 7, and 8, respectively, and those for Fmoc-L$_3$QG were 0.22, 0.38, 0.19, and 0.26 mM at pH 5, 6, 7, and 8, respectively.

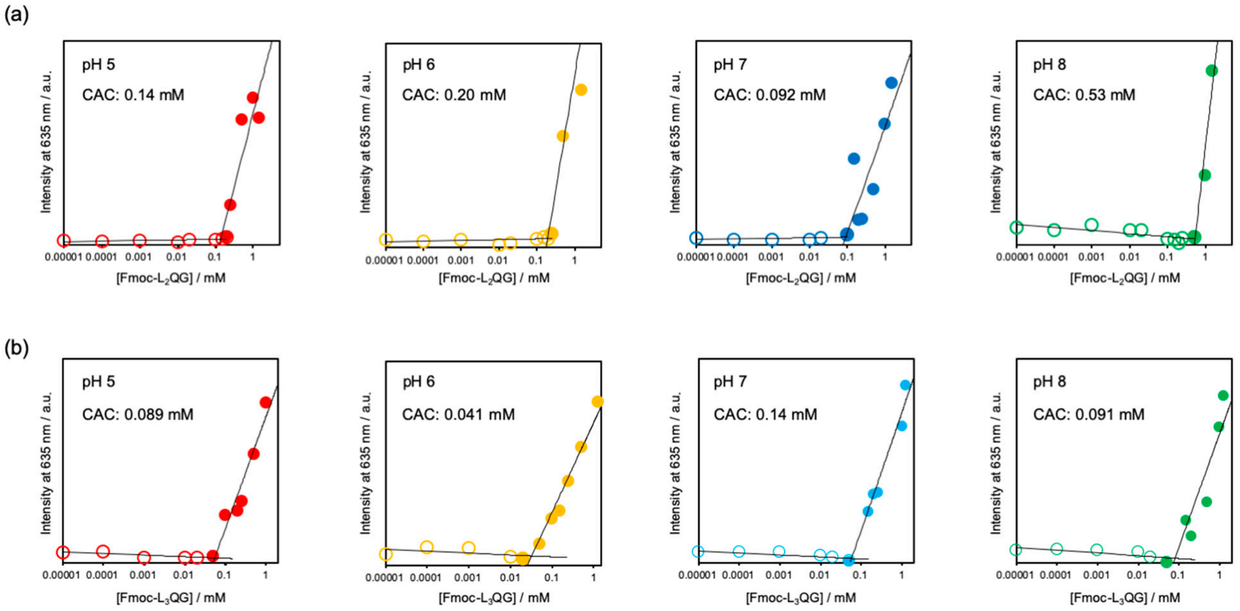

Figure S2. CAC plots of Fmoc-L$_2$QG (a) and Fmoc-L$_3$QG (b) at the pH 5-8.

3. Fluorescence spectra of Fmoc-L$_2$QG and Fmoc-L$_3$QG at pH 5-8 below CAC.

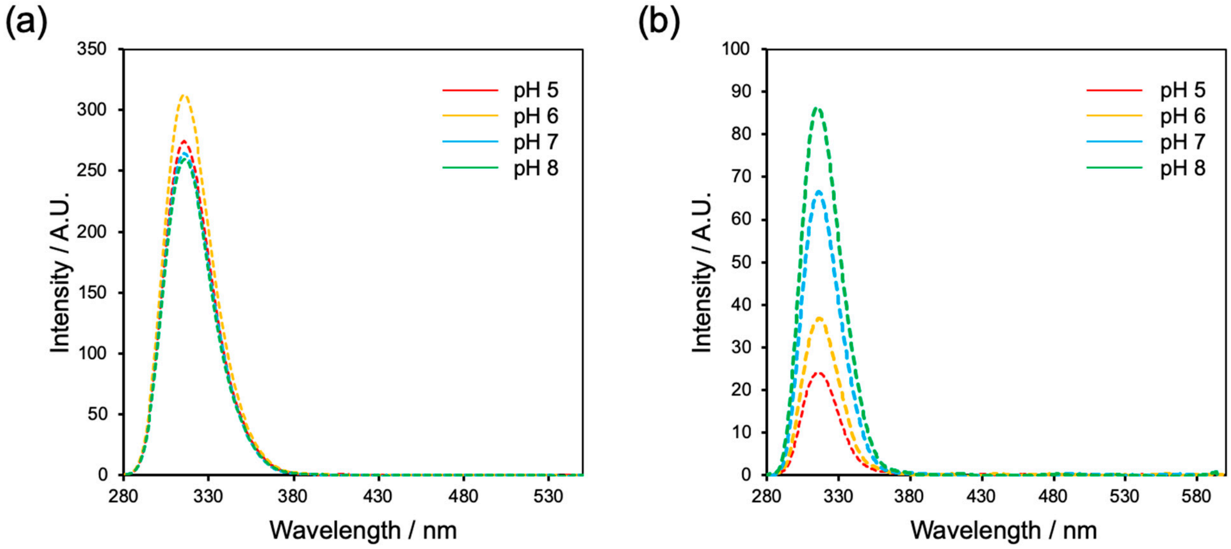

Figure S3. Fluorescence spectra of Fmoc-L$_2$QG (a) and Fmoc-L$_3$QG (b) at pH 5-8 below critical aggregation concentrations (CACs). λ_{ex} = 265 nm.

4. Evaluation of integration of Oregon green 488 cadaverine (OG) on Fmoc-L$_n$QG peptide assemblies by MALDI-TOF-MS.

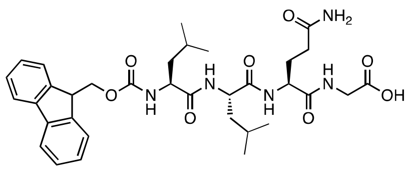

Fmoc-L$_2$QG
Chemical Formula: $C_{34}H_{45}N_5O_8$
Exact Mass: 651.33
Molecular Weight: 651.76

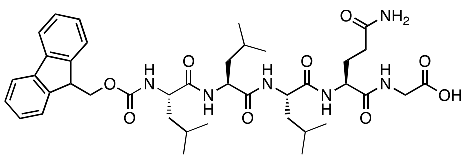

Fmoc-L$_3$QG
Chemical Formula: $C_{40}H_{56}N_6O_9$
Exact Mass: 764.41
Molecular Weight: 764.92

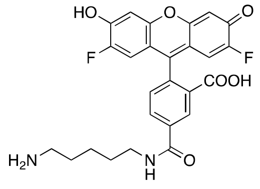

OG
Chemical Formula: $C_{26}H_{22}F_2N_2O_6$
Exact Mass: 496.14
Molecular Weight: 496.47

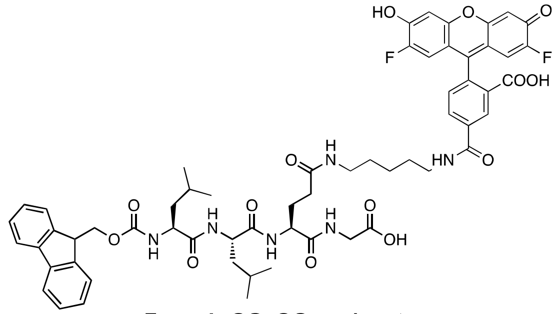

Fmoc-L$_2$QG–OG conjugate
Chemical Formula: $C_{60}H_{64}F_2N_6O_{14}$
Exact Mass: 1130.44
Molecular Weight: 1131.20

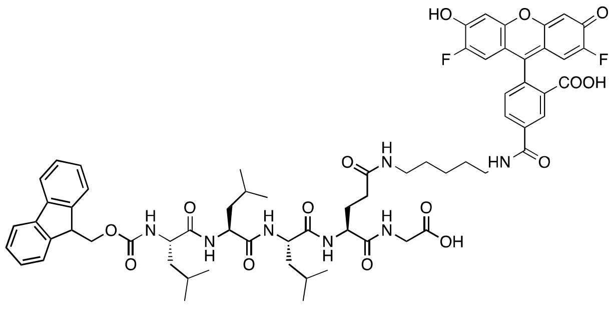

Fmoc-L$_3$QG–OG conjugate
Chemical Formula: $C_{66}H_{75}F_2N_7O_{15}$
Exact Mass: 1243.53
Molecular Weight: 1244.36

Scheme S1. Chemical structures, formula, and molecular weights of Fmoc-L$_n$QG peptides and their conjugate with OG.

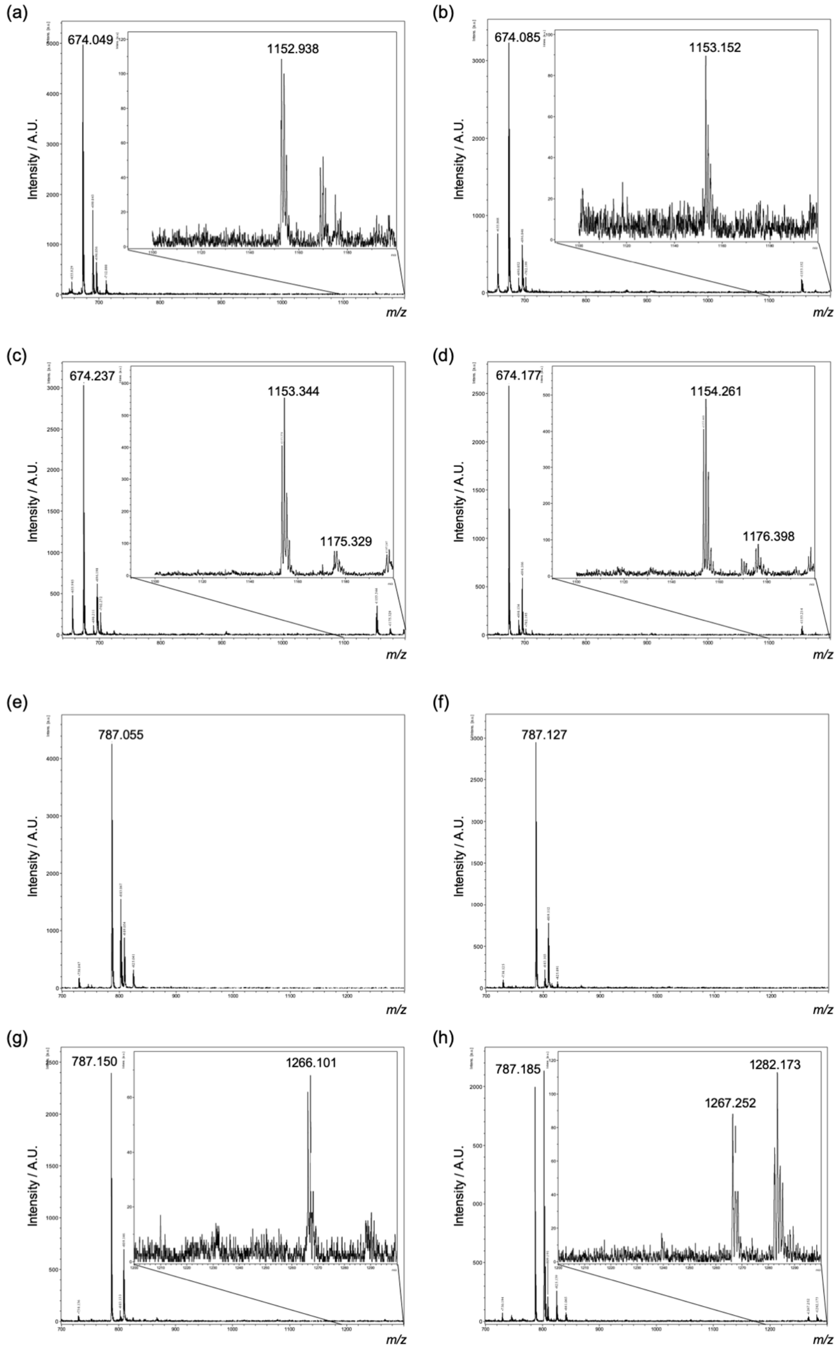

Figure S4. MALDI TOF MS spectra of Fmoc-L_2QG (a-d) and Fmoc-L_3QG (e-h) after the MTG reaction with OG at (a,e) pH 5, (b,f) pH 6, (c,g) pH 7 and (d,h) pH 8. MS peaks for Fmoc-L_nQG peptide–OG conjugates were detected except for Fmoc-L_3QG at pH 5 (e) and 6 (f), where reaction rates were too low to be detected by MALDI TOF MS.

5. Evaluation of MTG specific activity at various pH.

The specific activity of MTG was evaluated by a standard hydroxamate method [R1] using 10 mM (**Figure S5a**) or 200 mM (**Figure S5b**) buffers at various pH values. To prepare a calibration curve, hydroxamic acid was dissolved in Tris-acetate buffer (pH 6.0) to prepare a 40 mM solution, which was further diluted to prepare 2-, 4-, 8-, and 16-fold dilution series. MTG reaction was performed under the following conditions: [MTG] = 1 U/mL (determined by a standard method), [Z-QG] = 30 mM, [hydroxylamine] = 100 mM, 37°C, 10 min. The amount of hydroxamic acid formed was measured by measuring the absorbance at 525 nm after conversion of the hydroxamic acid to an iron complex under trichloroacetic acid conditions. The specific activity was calculated by defining the amount

of enzyme that produces 1 mol of hydroxamate per minute as the active unit of MTG, 1 U, and the relative specific activity of MTG was defined as the relative activity when the activity at pH 5 was set to 1. The specific activity at pH 6 and 7 using 200 mM buffer failed to be measured because precipitation was formed under these conditions (**Figure S5b**).

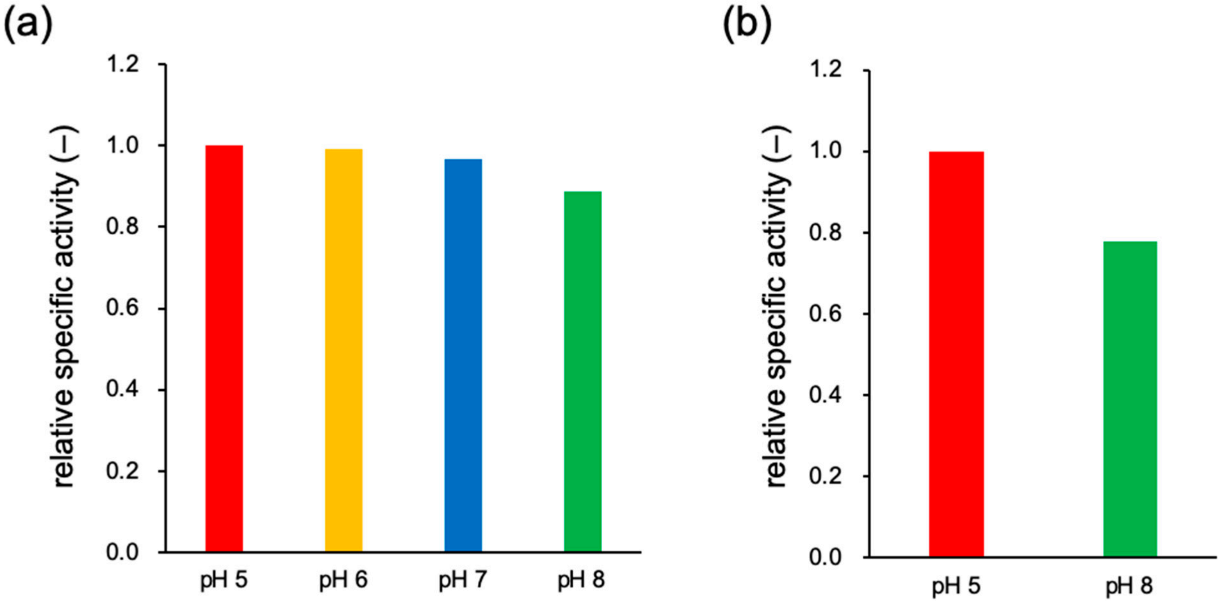

Figure S5. Relative specific activity of MTG measured using 10 mM (a) or 200 mM (b) buffers at various pH values.

6. MTG reaction of Fmoc-L$_n$QG peptides with TAMRA cadaverine and Sulforhodamine cadaverine.

A reaction sample for each self-assembled PA was prepared in 10 mM buffer at each pH ([Fmoc-L$_2$QG] = 2.0 mM, [Fmoc-L$_3$QG] = 1.0 mM) and MTG reaction was performed under the same conditions as OG except that TAMRA cadaverine (**Figure S6a,b**) or Sulforhodamine cadaverine (**Figure S6c,d**) was used instead of OG. The reaction proceeded at 25°C for 2 h, and the reaction was stopped by adding NEM to inactivate MTG. HPLC analysis (column: Inertsil ODS-3; eluent conditions: 0.1% TFA water/ACN from 60/40 to 20/80 linear gradient; flow rate: 1 mL/min) was conducted to evaluate the enzymatic reaction rate at each pH by using the value of an absorption (583 nm) from TAMRA cadaverine or Sulforhodamine cadaverine.

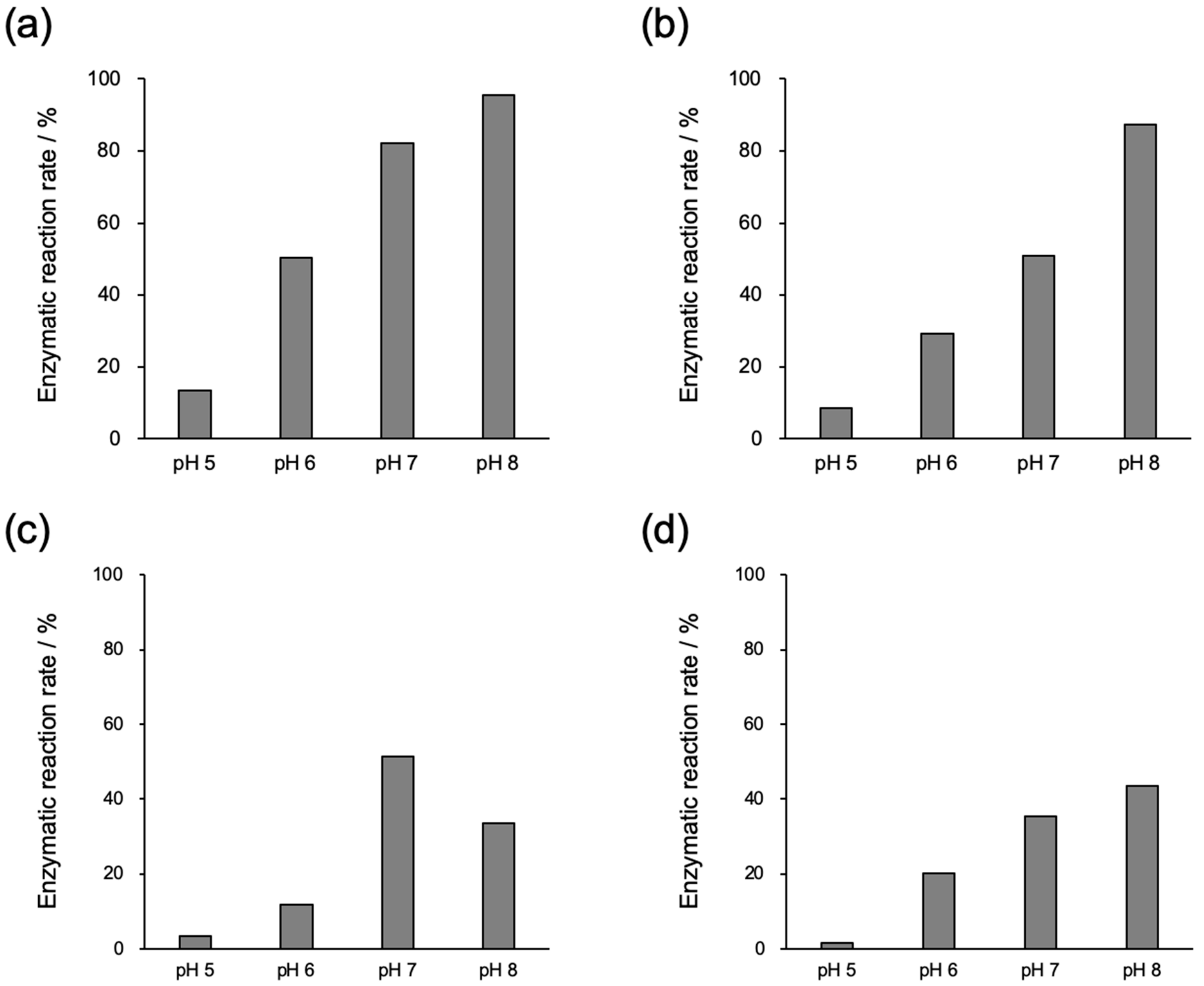

Figure S6. MTG enzymatic reaction rates of Fmoc-L$_2$QG (a,c) and Fmoc-L$_3$QG (c,d) with TAMRA cadaverine (a,b) or Sulforhodamine cadaverine (c,d).

7. MTG reaction rate of Fmoc-L$_n$QG with Ac-Lys-OH at concentrations below CAC.

Samples of monomeric Fmoc-L$_n$QG (50 µM) were prepared using 10 mM buffer at pH 5-8. Ac-Lys-OH (20 mM) and MTG (0.2 U/mL) were added, and MTG reaction was proceeded at 25 °C.

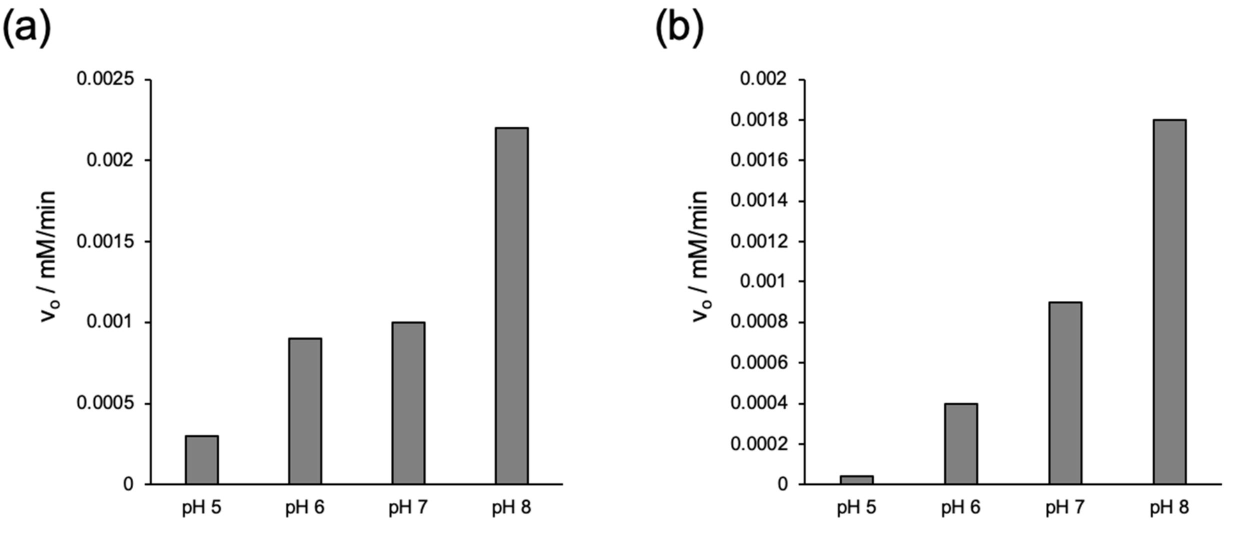

Figure S7. Initial reaction rates of monomeric Fmoc-L$_2$QG (a) and Fmoc-L$_3$QG (b).

8. MTG reaction rate of Z-QG with Ac-Lys-OH.

Z-QG (30 mM) were prepared using 200 mM buffers at pH 5-8. Ac-Lys-OH (100 mM) and MTG (0.3 U/mL) were added, and MTG reaction was proceeded at 37°C.

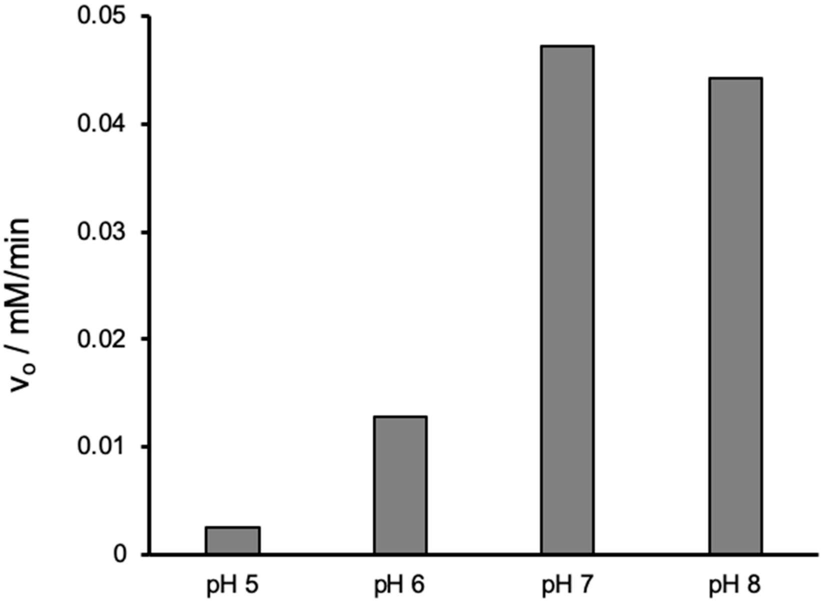

Figure S8. Initial reaction rates of Z-QG.

9. Titration curves of Fmoc-L$_n$QG assemblies to determine apparent pKa values.

One milliliter of 2.5 mM Fmoc-L$_2$QG or 1.5 mM Fmoc-L$_3$QG solution was prepared using ultrapure water. Sodium hydroxide (0.01 M) was added to the above Fmoc-L$_n$QG solutions until the solution pH reached to 10.0. The solutions were vortexed until the peptides were fully dissolved. The titration experiments were performed by adding small volumes of 0.01 M HCl.

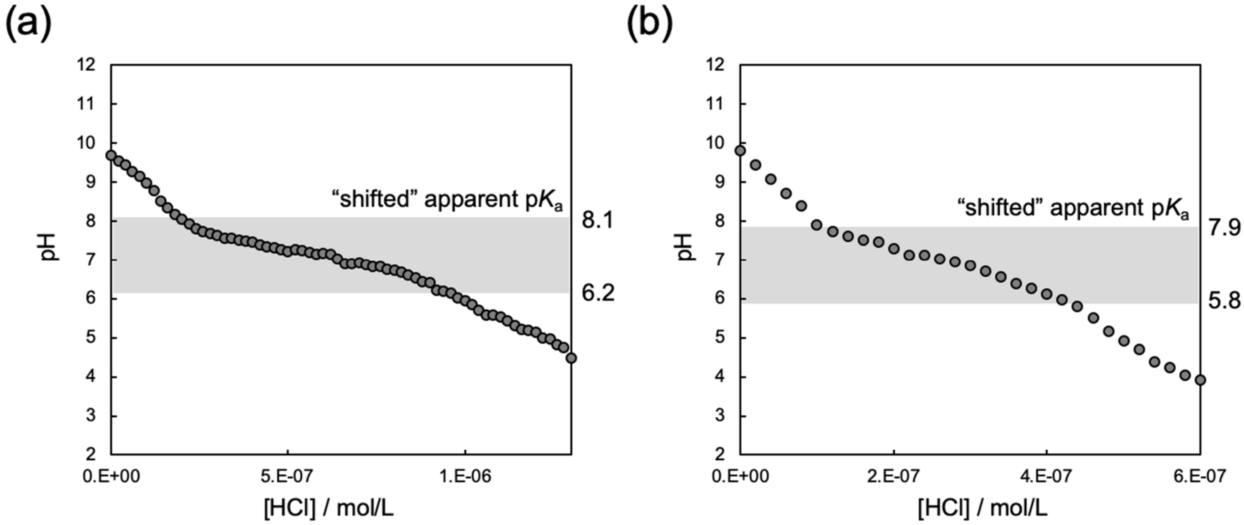

Figure S9. Titration curves of Fmoc-L$_2$QG assembly (a) and Fmoc-L$_3$QG assembly (b).

Reference

1. Folk, J.E.; Cole, P.W. Mechanism of Action of Guinea Pig Liver Transglutaminase. *J. Biol. Chem.* **1966**, *241*, 5518–5525, doi:10.1016/s0021-9258(18)96373-8.
